# Local Postsynaptic Signaling on Slow Time Scales in Reciprocal Olfactory Bulb Granule Cell Spines Matches Asynchronous Release

**DOI:** 10.3389/fnsyn.2020.551691

**Published:** 2020-11-16

**Authors:** Tiffany Ona Jodar, Vanessa Lage-Rupprecht, Nixon M. Abraham, Christine R. Rose, Veronica Egger

**Affiliations:** ^1^Regensburg University, Regensburg, Germany; ^2^Institut D’Investigacions Biomèdiques, Barcelona, Spain; ^3^Fraunhofer Institute for Algorithms and Scientific Computing, St. Augustin, Germany; ^4^Indian Institute of Science Education and Research, Pune, India; ^5^Heinrich Heine University Duesseldorf, Düsseldorf, Germany

**Keywords:** olfactory bulb, granule cell, two-photon (2P) uncaging, two-photon sodium imaging, two-photon calcium imaging, asynchronous release, reciprocal synapse, recurrent inhibition

## Abstract

In the vertebrate olfactory bulb (OB), axonless granule cells (GC) mediate self- and lateral inhibitory interactions between mitral/tufted cells via reciprocal dendrodendritic synapses. Locally triggered release of GABA from the large reciprocal GC spines occurs on both fast and slow time scales, possibly enabling parallel processing during olfactory perception. Here we investigate local mechanisms for asynchronous spine output. To reveal the temporal and spatial characteristics of postsynaptic ion transients, we imaged spine and adjacent dendrite Ca^2 +^- and Na^+^-signals with minimal exogenous buffering by the respective fluorescent indicator dyes upon two-photon uncaging of DNI-glutamate in OB slices from juvenile rats. Both postsynaptic fluorescence signals decayed slowly, with average half durations in the spine head of t_1__/__2__Δ[Ca^2 +^]_i_ ∼500 ms and t_1__/__2__Δ[Na^+^]_i_ ∼1,000 ms. We also analyzed the kinetics of already existing data of postsynaptic spine Ca^2 +^-signals in response to glomerular stimulation in OB slices from adult mice, either WT or animals with partial GC glutamate receptor deletions (NMDAR: GluN1 subunit; AMPAR: GluA2 subunit). In a large subset of spines the fluorescence signal had a protracted rise time (average time to peak ∼400 ms, range 20 to >1,000 ms). This slow rise was independent of Ca^2 +^ entry via NMDARs, since similarly slow signals occurred in ΔGluN1 GCs. Additional Ca^2 +^ entry in ΔGluA2 GCs (with AMPARs rendered Ca^2 +^-permeable), however, resulted in larger ΔF/Fs that rose yet more slowly. Thus GC spines appear to dispose of several local mechanisms to promote asynchronous GABA release, which are reflected in the time course of mitral/tufted cell recurrent inhibition.

## Introduction

In the vertebrate olfactory bulb (OB), the lateral dendrites of the principal mitral and tufted cells are interconnected via local GABAergic interneurons. The most abundant class of these local neurons, the axonless granule cells (GC), mediate self- and lateral inhibitory interactions between mitral/tufted cells via reciprocal dendrodendritic synapses that on the GC dendrite are housed in large spines (Shepherd, 2004). These reciprocal synapses have been directly demonstrated to play a role in odor discrimination and learning (Abraham et al., 2010; Gschwend et al., 2015). Moreover, they are also critically involved in generating bulbar γ-oscillations (Rall and Shepherd, 1968; Nusser et al., 2001; Lagier et al., 2004), which are thought to contribute to odor coding via synchronization and gating of mitral cell output (e.g., Buonviso et al., 2003; Fukunaga et al., 2014; Osinski and Kay, 2016).

Recordings of dendrodendritic inhibition of mitral cells have revealed that recurrent inhibition happens as a barrage of IPSCs (Isaacson and Strowbridge, 1998; Schoppa et al., 1998). Within this barrage, early IPSCs will occur with a very short latency (below 10 ms), but recurrent activity takes several hundreds of milliseconds to subside. While this long tail of recurrent inhibition is unlikely to directly contribute to odor discrimination itself (Uchida and Mainen, 2003; Abraham et al., 2004, 2010), it may well play a role in learning and memory formation (Gschwend et al., 2015; see section “Discussion”).

The underlying asynchronous release is at least to a major extent due to processing in GCs, since asynchronous responses were demonstrated following flash photolysis of Ca^2 +^ in mitral cell lateral dendrites (Chen et al., 2000). Moreover, while the massive release of glutamate during the commonly used protocol for mitral cell excitation (20–50 ms depolarization in the voltage-clamp mode) might result in activation of slow release pathways not accessible to unitary transmission, we have shown recently, that local, unitary-like two-photon uncaging of glutamate (TPU) can still cause prolonged release of GABA within a time window of up to 500 ms post uncaging (Lage-Rupprecht et al., 2020).

As to possible mechanisms for late output, unitary EPSPs evoked by spontaneous mitral/tufted cell input or local TPU are mediated by both AMPA and NMDA receptors, and decaying with a time constant <50 ms (as recorded at the GC soma, Bywalez et al., 2015). Thus slower actions downstream of ionotropic receptors would be required to trigger cascades that result in asynchronous release events beyond 100 ms. A number of global mechanisms has been proposed to promote asynchronous release from GC spines. These include a delay of global GC action potentials (AP) due to the prominent I_A_ current (Schoppa and Westbrook, 1999; Kapoor and Urban, 2006), and a prolonged Ca^2 +^ entry due to synaptic activation of a non-specific cation current I_CAN_, possibly in coincidence with global APs (Hall and Delaney, 2002; Egger, 2008; Stroh et al., 2012).

Since as in other synapses reciprocal release of GABA is Ca^2 +^-dependent (Isaacson and Strowbridge, 1998), how could spine Ca^2 +^ signals mediate asynchronous release? To answer this question, we explored several potential local mechanisms that might be involved in slow spine Ca^2 +^ signaling and thus are directly related to the biophysical properties of individual spines.

While the endogenous Ca^2 +^ buffering capacity κ_*E*_ in GC spines is not unusually high (∼120) and thus cannot explain lingering Ca^2 +^, the Ca^2 +^ extrusion from the spine cytosol is sluggish (rate γ∼500 s^–1^ at RT), which might support asynchronous output (Egger and Stroh, 2009).

As to postsynaptic spine Ca^2 +^ signals upon glomerular mitral cell stimulation (100 μM OGB-1, Egger et al., 2005), responses in juvenile rat GC spines are robust, with an average amplitude of ∼40% ΔF/F, and rise within ∼80 ms. Their decay kinetics are slower than those of backpropagating AP-mediated transients, with a clearly bimodal distribution of durations. While ∼2/3 of spine signals decayed by half within ∼600 ms, the remaining 1/3 decayed very slowly, with half durations beyond 1.5 s. Identical signal properties including the fraction of “slow spines” are observed in response to TPU of glutamate (Bywalez et al., 2015). This unitary postsynaptic Ca^2 +^ entry is mainly mediated by NMDA receptors, with additional contributions by low- and high voltage activated Ca^2 +^ channels and Ca^2 +^-induced Ca^2 +^ release (CICR; Egger et al., 2005). Relief from the Mg^2+^ block is provided via a local AP (“spine spike,” Bywalez et al., 2015), while native GC AMPA receptors are not Ca^2 +^-permeable (Jardemark et al., 1997; Egger et al., 2005).

However, these previous recordings of synaptic fluorescence transients were all performed with the Ca^2 +^ indicator dye OGB-1 (100 μM, K_d_ = 200 nM). Therefore transients are substantially buffered and do not reflect true kinetics of Ca^2 +^ signals (Egger and Stroh, 2009), even though their observed decay times seem to fit well with the time scale of asynchronous release. Thus here we asked whether synaptic signals are indeed slow, also in comparison to AP-mediated transients, by using a low-affinity Ca^2 +^ dye.

Moreover, we had observed earlier on that the triggering of long-lasting depolarizations in the wake of synaptically evoked GC APs required both NMDA receptor activation and opening of voltage-gated Na^+^ channels (Na_v_; observed in both juvenile rats and adult mice) and that these long-lasting depolarizations were carried by TRPC1/4 heteromeric channels (Egger, 2008; Stroh et al., 2012). Since we know by now that local postsynaptic inputs can trigger “spine spikes” within the spine head (Bywalez et al., 2015), we hypothesized that already local synaptic signals might involve a long-lasting cationic inward current I_CAN_ (via TRPCs or otherwise). To this end, we combined Na^+^ imaging based on the dye SBFI (Rose et al., 1999; Ona-Jodar et al., 2017) with TPU of glutamate.

Finally, we noticed that in spite of similar endogenous Ca^2 +^ dynamics and similar amplitudes of spontaneous and evoked synaptic transients in adult mouse and juvenile rat GCs (Egger et al., 2005; unpublished observations Egger and Stroh, Egger and Stroh, 2009; Abraham et al., 2010), postsynaptic Ca^2 +^ signals in adult mouse GCs were yet slower with regard to their rise time (original data set from Abraham et al., 2010, in which kinetics had not been analyzed quantitatively). This study had revealed a correlation between behavioral performance in a go/no-go odor discrimination task, and modifications of postsynaptic ΔCa^2 +^ into the majority of GC spines via viral transfection, across three sample groups: (1) WT mice, (2) mice with a deletion of the GluA2 AMPAR subunit (ΔGluA2; increased Ca^2 +^ entry) which resulted in faster discrimination and thus a gain of function, and (3) mice with a deletion of the NR1 NMDAR subunit (ΔGluN1, i.e., reduced Ca^2 +^ entry), which resulted in slowed discrimination, i.e., a loss of function. Here we provide a quantitative analysis of the kinetics of the respective Ca^2 +^ signals and the response probability and use the genetic pharmacology provided by the viral knockdown to infer possible mechanisms for the slow rise.

In summary, here we aim to unravel the pyhsiological time courses of postsynaptic Ca^2 +^ and Na^+^ signals in juvenile rat GCs in order to investigate their overlap with the previously established time course of asynchronous release. Moreover, we describe an additional potential source of delayed release from adult mouse GC spines.

## Methods

### Juvenile Rat Experiments: Preparation, Electrophysiology

Sagittal OB brain slices (thickness 300 μm) were prepared in artificial cerebrospinal fluid (ACSF, composition see below) following procedures in accordance with the rules laid down by the EC Council Directive (86/89/ECC) and German animal welfare legislation. Slices were incubated a water bath at 33°C for 30 min and then kept at room temperature (22°C) until recordings were performed.

The extracellular ACSF was bubbled with carbogen and contained (in mM): 125 NaCl, 26 NaHCO_3_, 1.25 NaH_2_PO_4_, 20 glucose, 2.5 KCl, 1 MgCl_2_, and 2 CaCl_2_. Whole cell current clamp recordings were performed at room temperature (22 °C) and granule cells were held near their resting potential of −80 mV. Granule cells were filled with an internal solution containing the following substances (in mM): 130 K-Methylsulfate, 10 HEPES, 4 MgCl_2_, 2 ascorbic acid, 10 phosphocreatine-di-tris-salt, 2.5 Na_2_ATP, 0.4 NaGTP, and 1 mM SBFI (Na^+^-binding benzofuran isophthalate, Teflabs, Austin, TX, United States and Molecular Probes, Eugene, OR, United States) or 0.1 OGB-6F (Ca^2 +^ indicator, Thermo Fisher Scientific, Waltham, MA, United States). The patch pipette resistance varied between 6 and 7 MΩ.

### Juvenile Rat Experiments: Combined Two-Photon Imaging and Uncaging

For Na^+^ imaging experiments, electrophysiology and imaging were performed as in Ona-Jodar et al. (2017), and for Ca^2 +^ imaging experiments as in Bywalez et al. (2015). Uncaging is also described in detail in Bywalez et al. (2015). Imaging and uncaging were performed on a Femto-2D-uncage microscope (Femtonics, Budapest, Hungary). Two tunable, Verdi-pumped Ti:Sa lasers (Chameleon Ultra I and II respectively, Coherent, Santa Clara, CA, United States) were used in parallel. The first laser was set either to 840 nm for excitation of OGB-6F or to 800 nm for excitation of SBFI in GC spines and dendrites, and the second laser was set to 750 nm for uncaging of caged glutamate. The two laser lines were directly coupled into the pathway of the microscope with a polarization cube (PBS102, Thorlabs Inc., Newton, NJ, United States) and two motorized mirrors. As caged compound we used DNI-caged glutamate (DNI; Femtonics). DNI was used at 1 mM in a closed perfusion circuit with a total volume of 12 ml. Caged compounds were washed in for at least 10 min before starting measurements. The uncaging laser was switched using an electro-optical modulator (Pockels cell model 350-80, Conoptics, Danbury, CT, United States).

Na^+^ and Ca^2 +^ signals were imaged in line scanning mode with a temporal resolution of ∼1 ms. The scan position was checked and readjusted if necessary before each measurement to account for drift.

### Adult Mouse GC Ca^2 +^ Imaging (Data From Abraham et al., 2010)

The experiments in adult mice are described in Abraham et al. (2010); the new analyses presented here are based on the very same data set. Briefly (see Abraham et al., 2010, for details), GC-specific deletion of GluA2 AMPAR subunit and GluN1 NMDAR subunit had been achieved by viral expression of Cre recombinase in mice with conditional alleles of GluA2 and GluN1. To restrict the deletion to GCs we had injected rAAV Cre only in the anterior portion with respect to the center of the dorsal OB surface. OB slices had been prepared after an incubation time of at least 2 weeks. GluA2- and GluN1-depleted GCs had been identified by somatic fluorescence arising from co-expression of Cre recombinase and Kusabira orange (Tang et al., 2009). Mitral/tufted cells had been activated via glomerular extracellular stimulation, and responding GC spines had been searched for with two-photon Ca^2 +^ imaging in GCs patched below the stimulated glomerulus that had responded to glomerular stimulation with a detectable EPSP (see also Figure 3A).

### Data Analysis and Statistics

Imaging data were analyzed with custom written macros in Igor Pro (Wavemetrics, Lake Oswego, OR, United States), as described previously (Egger et al., 2003, 2005). All imaging signals (OGB-6, OGB-1, SBFI) were analyzed in terms of ΔF/F = (F(t) − F_0_)/F_0_. Rise times were measured between 20 and 80% of the absolute maximal ΔF/F amplitude, and half durations t_1__/__2_ reflect the period from this maximal amplitude to the half-maximal amplitude. SBFI ΔF/F signals were converted into absolute concentration changes Δ[Na^+^]_i_ according to the previously established calibration on the same system: for non-saturating signals a 10% change in fluorescence emission of SBFI corresponds to a change of 22.3 mM in [Na^+^]_i_ (Ona-Jodar et al., 2017). The response probability is an estimate of the release probability and was calculated as the ratio of detected responses to the total number N of stimulations (average *N* = 14 ± 5 in WT).

Statistical comparisons were made with non-parametric tests (Wilcoxon test for paired and Mann-Whitney test for unpaired data sets). Comparisons between WT GC responses and the ΔGluA2 and ΔGluN1 GC groups were made via pairwise Mann-Whitney tests with Bonferroni correction. Frequency distributions of parameters were compared with the Kolmogorov-Smirnov test. Mean values are given ± SD.

## Results

### Time Course of Synaptic Spine Ca^2 +^ Signals With Minimal Exogenous Buffering

To investigate the local mechanisms underlying the asynchronous component of reciprocal GABA release, we aimed to detect local postsynaptic Ca^2 +^ signaling in GC spines with as little exogenous buffering as possible, since sluggish extrusion of Ca^2 +^ might also contribute to delayed release (Egger and Stroh, 2009). The low-affinity dye OGB-6F (K_d_ ≈ 8 μM, Tran et al., 2018) was used at a concentration of 100 μM, where the kinetics of OGB-6F fluorescence transients in response to single somatic APs (ΔF/F)_sAP_ are identical to the kinetics determined by extrapolation of measurements with varying concentrations of OGB-1 (K_d_ ≈ 0.2 μM) to zero added buffer (Egger and Stroh, 2009).

**FIGURE 1:**
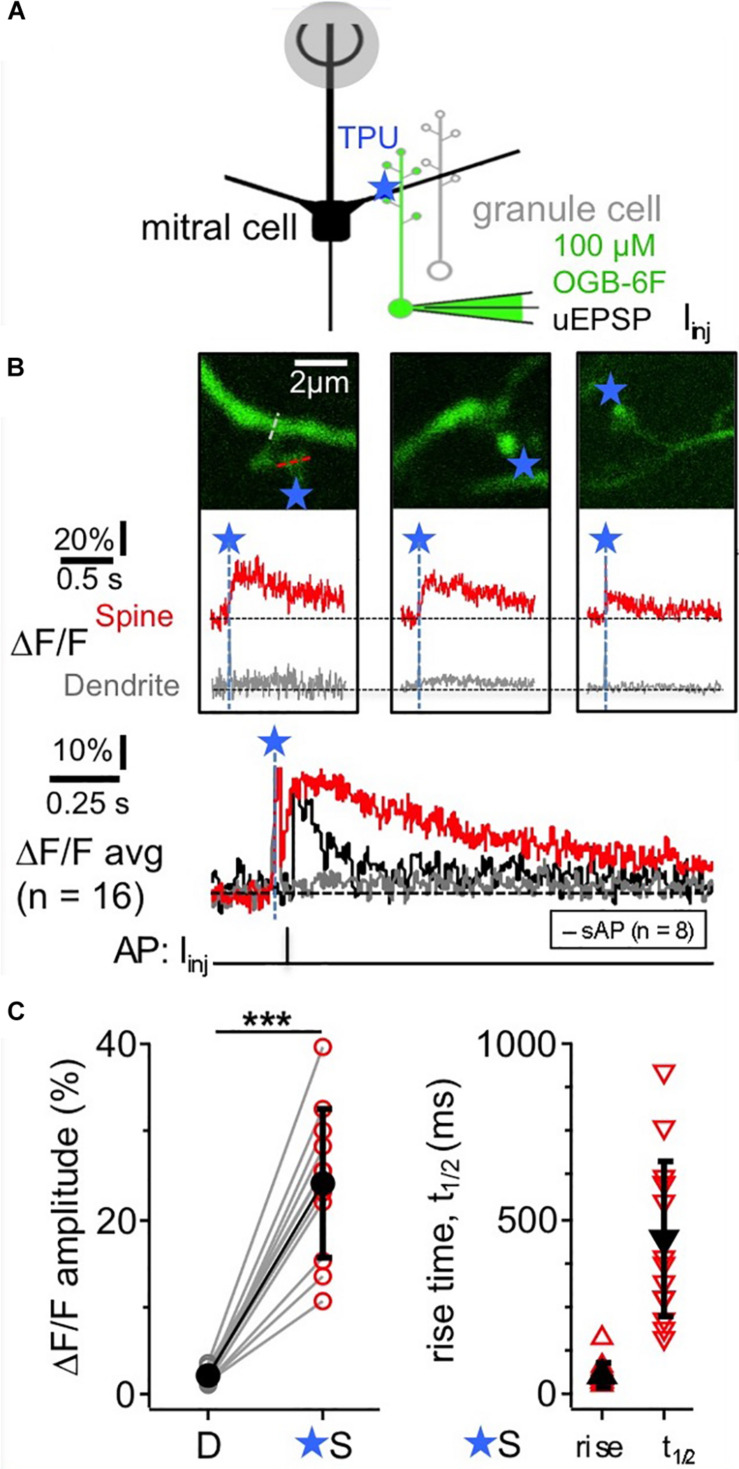
TPU-evoked Ca^2 +^ entry into juvenile rat GC spines with low exogenous buffering. Imaging of TPU-evoked Ca^2 +^ transients within GC spines with low exogenous buffering (100 μM OGB-6F). **(A)** Scheme of experiment: whole cell recording from GC (filled with dye via pipette) and TPU at spine head. The pipette was also used for brief current injections to evoke somatic APs (sAP). **(B)** Top: two-photon scans (z-projections) of three representative examples of individual spines filled with OBG-6F. Blue stars denote uncaging locations. Red and gray dotted line indicate line scan positions. Middle: respective averaged fluorescence transients (ΔF/F)_TPU_ that were collected from line scans across the spine heads above (S, red) and the adjacent dendrite at the base of the spine neck (D, gray). Blue dashed lines and star: time point of uncaging. Bottom: (ΔF/F)_TPU_ transients averaged across experiments (Ca^2 +^ imaging: *n* = 11 spines) with the same time axis, spine response in red and dendrite response in gray. The black trace in the Ca^2 +^ imaging graph represents the averaged response (ΔF/F)_AP_ to a backpropragating somatically evoked AP (recorded in *n* = 8 of the 11 spines). **(C)** Cumulative plots of (ΔF/F)_TPU_ amplitudes in dendrite and spine pairs (highly significantly different: *P* < 0.001, Wilcoxon test), and of rise times and half durations t_1__/__2_ of (ΔF/F)_TPU_ within the spine heads (mostly not detectable in the dendrites).

TPU of DNI with similar parameters as in Bywalez et al. (2015; Figure 1A) evoked Ca^2 +^ transients (ΔF/F)_TPU_ in juvenile rat GC spine heads (postnatal days PND11-19), with a mean amplitude of 24 ± 9%, a mean rise time of 55 ± 32 ms and a mean half duration t_1__/__2_ of 445 ± 225 ms (*n* = 11 spines, Figures 1B,C). These transients were strictly localized to the spine head (mean (ΔF/F)_TPU_ amplitude in adjacent dendritic shaft 2 ± 1%, ratio vs. spine head 0.09 ± 0.03). While t_1__/__2_ was difficult to analyze in some of the individual spine responses because of noise, the averaged transient yielded a t_1__/__2_ of ∼550 ms, substantially slower than the half-duration of AP-mediated transients recorded in a subset of these spines (*n* = 8, t_1__/__2_ of averaged ΔF/F ∼100 ms, Figure 1B bottom). The influence of buffering on the rise time should be less pronounced since the latter mostly reflects the duration of Ca^2 +^ entry into the cytoplasm. Indeed, the set of rise times is statistically not different from a previous TPU data set using also DNI and OGB-1 (rise time 76 ± 57 ms, median 60 ms, *n* = 42, *P* = 0.19, Mann-Whitney test; data from Bywalez et al., 2015).

### Time Course of Synaptic Spine Na^+^ Signals With Minimal Added Exogenous Buffering

**FIGURE 2:**
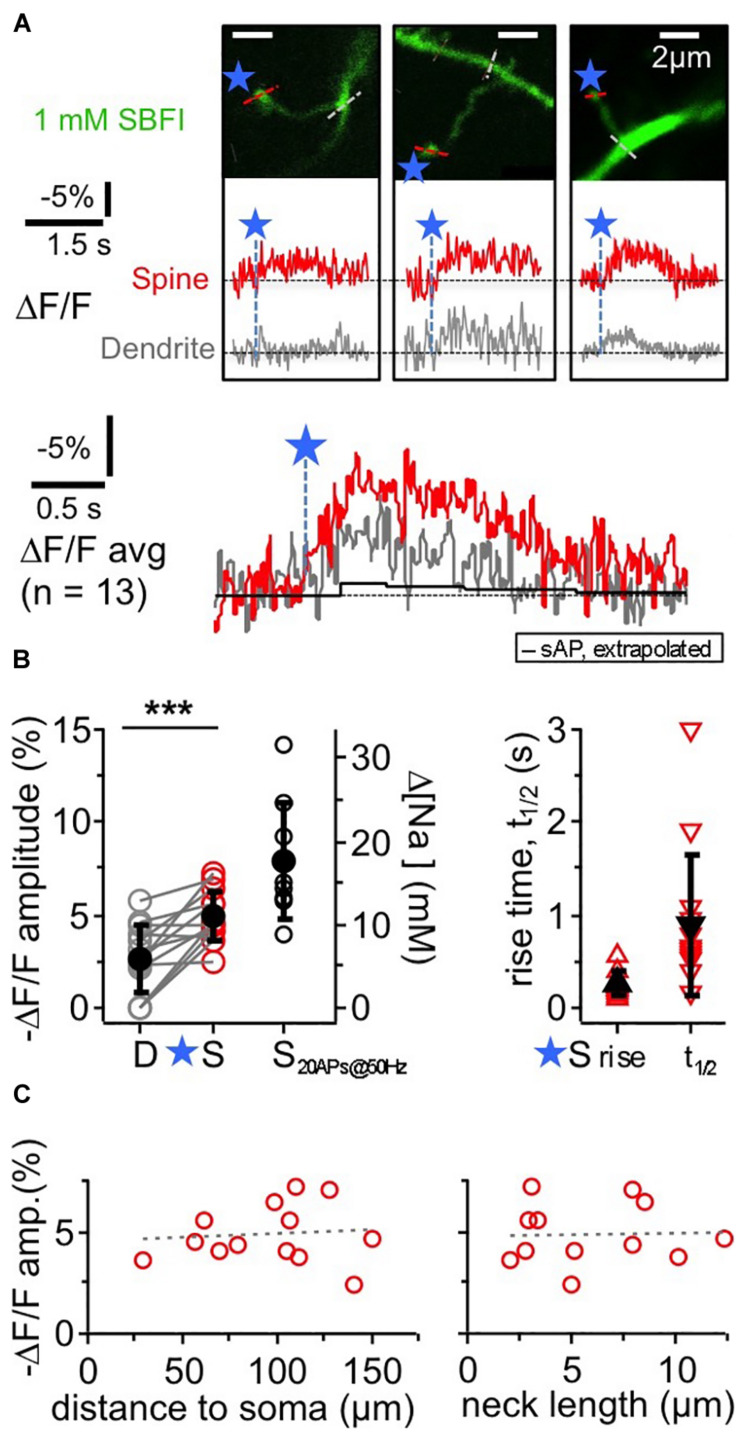
TPU-evoked Na^+^ entry into juvenile rat GC spines. Imaging of TPU-evoked Na^+^ transients within GC spines with low exogenous buffering (1 mM SBFI). Experimental setup as in Figure 1A. **(A)** See also Figure 1B. Top: two-photon z-stacks of three representative examples of individual spines filled with SBFI. Blue stars denote uncaging locations. Red and gray dotted line indicate line scan positions for the leftmost spine. Middle: respective averaged fluorescence transients (ΔF/F)_TPU_ within the above spines (S, red) and within the adjacent dendrite (at the base of the spine neck, D, gray). Bottom: (ΔF/F)_TPU_ transients averaged across experiments (*n* = 13 spines) with the same time axis, spine response in red and dendrite response in gray. Note the similar time course of the dendritic response. Black trace: extrapolated response (ΔF/F)_AP_ to a single backpropragating somatically evoked AP (from mean response to 20 APs at 50 Hz, see **(B)** Amplitude (ΔF/F)_AP_ ≈ –0.4%, t_1__/__2_ ≈ 1.7 s). **(B)** Cumulative plots. Left panel: (ΔF/F)_TPU_ amplitudes in dendrite and spine pairs (highly significantly different, *P* < 0.001, Wilcoxon test) and (ΔF/F)_5__0H__z_ amplitudes in response to a 50 Hz train of 20 APs in a subset of the same spine heads (*n* = 9). Right axis: changes in [Na^+^]_i_ concentration (calibration from Ona-Jodar et al., 2017). Right panel: half durations t_1__/__2_ of (ΔF/F)_TPU_ within the spine heads (t_1__/__2_ could not be measured in most dendritic transients because of noise). **(C)** (ΔF/F)_TPU_ spine amplitudes vs. distance of the spine to the soma and spine neck length (means 99 ± 35 μm and 5.9 ± 3.3 μm respectively, *n* = 12). Dashed lines: linear fit. No significant correlations.

Postsynaptic Na^+^ signals could report the activity of the Ca^2 +^-impermeable GC AMPARs and of spine Na_v_s and TRPC1/4 in a more direct way than Ca^2 +^ signals and thus yield additional information on the state of the locally activated GC spine. We performed two-photon Na^+^ imaging using SBFI at a concentration of 1 mM. This is far below both the Na^+^ concentration of 15 mM in the internal solution and the apparent K_D_ of SBFI, so the degree of introduced buffering is negligible (Mondragão et al., 2016; Figure 2).

The ensuing Na^+^ signals following TPU of glutamate at individual spine heads with similar parameters as in the OGB-6F experiments had a mean amplitude of −(ΔF/F)_TPU_ = 4.9 ± 1.4% in the spine head of juvenile rat GCs (PND 11–18). They were localized to the spine head to some extent but mostly also detectable in the adjacent dendritic shaft (mean amplitude ratio dendrite/spine 0.56 ± 0.38 of spine signal; *P* < 0.001 vs. spine signal amplitude; *n* = 13 spines in 11 GCs). Conversion of the spine signal amplitude to absolute changes in [Na^+^]_i_ (Rose et al., 1999; Ona-Jodar et al., 2017) yielded a mean increase Δ[Na^+^]_i_ by ∼10 mM. The average rise time was 250 ± 130 ms and the half duration t_1__/__2_ = 890 ± 770 ms in the spines, including frequently observed plateau-like phases. Individual (ΔF/F)_TPU_ signals in dendritic shafts were usually too noisy for kinetic analysis.

As for TPU-evoked Ca^2 +^ transients (Bywalez et al., 2015), there were no significant correlations between spine (ΔF/F)_TPU_ amplitudes and distance to soma or spine neck length (Figure 2C), and also no correlation between the amplitude ratio of spine/dendrite and spine neck length (not shown).

Again, we averaged data across all spine/dendrite pairs (Figure 2A bottom). The averaged spine signal showed an initial plateau-like phase of ∼600 ms, and the averaged dendrite signal mirrored the kinetics of the spine signal, which is expected because of the fast diffusion of Na^+^ into the dendrite (Mondragão et al., 2016). Still, these TPU-evoked Na^+^ signals are very slow in view of the overall fast diffusion of Na^+^ and also compared to recent data from synaptic Na^+^ signals in hippocampal pyramidal neuron spines (their t_1__/__2_ ∼20 ms; Miyazaki and Ross, 2017), and therefore are best explained by a persistent influx of Na^+^ (see section “Discussion”). The TPU-evoked spine Na^+^ signals are also very large as compared to the Na^+^ influx induced by single backpropagating APs. The latter was on the order of 0.4 mM (as extrapolated from train stimulation of a subset of the same spines with 20 APs at 50 Hz: (ΔF/F)_5__0H__z_ = −7.9 ± 3.1% *n* = 9, Figure 2B; see Figure 5 in Ona-Jodar et al. (2017) for train responses).

From these experiments we conclude that the time course of asynchronous components of GABA release triggered by unitary activation (Lage-Rupprecht et al., 2020) matches well with substantial and prolonged elevation of postsynaptic Na^+^ and Ca^2 +^ concentrations in the GC spine. Late release therefore might result from local processing following unitary inputs to the reciprocal spine (see section “Discussion”).

### Postsynaptic GC Spine Ca^2 +^ Signals in Adult Mice

As shown above, both postsynaptic Ca^2 +^ and Na^+^ signaling in juvenile rat GC spines is likely to persist for several 100 ms. Moreover, we noticed that in spite of similar endogenous Ca^2 +^ dynamics (with regard to both buffering capacity and extrusion: Egger and Stroh, 2009), postsynaptic spine Ca^2 +^ transients in adult mice evolved yet more sluggishly. Here we analyzed the kinetics and response probability of postsynaptic GC spine Ca^2 +^ signals in response to glomerular stimulation from an earlier data set that was recorded with two-photon fluorescence imaging in acute bulb slices from WT animals or from animals with partial GC GluN1 or GluA2 deletions via viral transfection (PND 36–66; dye 100 μM OGB-1; Abraham et al., 2010).

**FIGURE 3:**
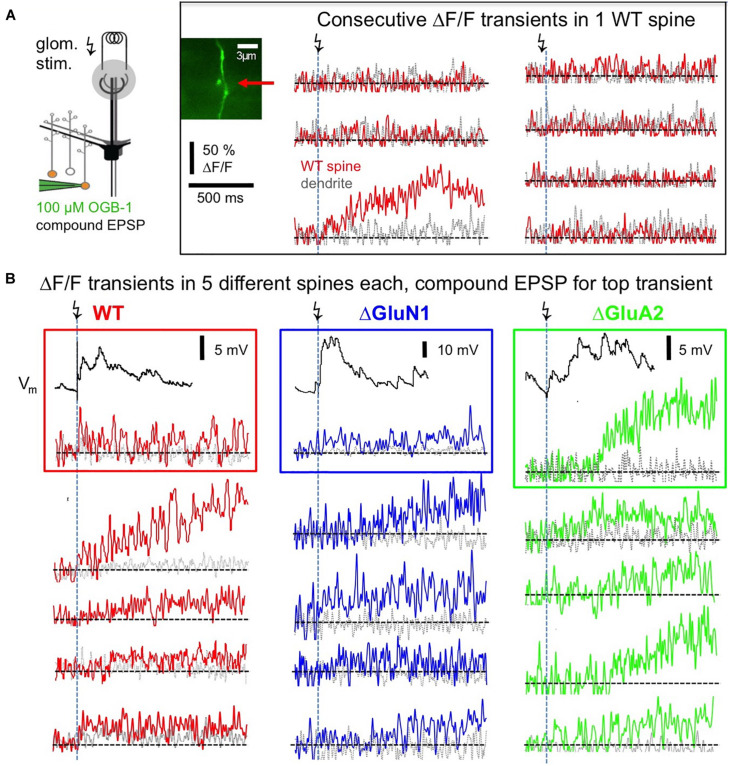
Postsynaptic responses in adult WT, ΔGluA2 and ΔGluN1 GC spines. **(A)** Left: Scheme of experiment (modified from Abraham et al., 2010, their Figure 2). Electrical glomerular stimulation and whole-cell recording of the resulting compound EPSP from a GC, including two-photon imaging of a responding spine and its adjacent dendritic shaft. Genetically modified GCs (ΔGluA2 and ΔGluN1) were identified by the expression of the fluorescent construct Kusabira-Orange. Middle: scan of a WT spine and dendrite. Right: line scans through the spine and dendrite during consecutive glomerular stimulations. Only one response occurred (3rd trace), that shows a slowly evolving Ca^2 +^ transient confined to the spine head. **(B)** Single responses from other spines, with each trace imaged in a different spine. Same scales for ΔF/F and time as in **(A)**. Top transients with their associated compound EPSP recordings. Left (red): WT GCs. Middle (blue): ΔGluN1 GCs. Right (green): ΔGluA2 GCs. Note the larger size and yet slower evolution compared to WT and ΔGluN1.

**FIGURE 4:**
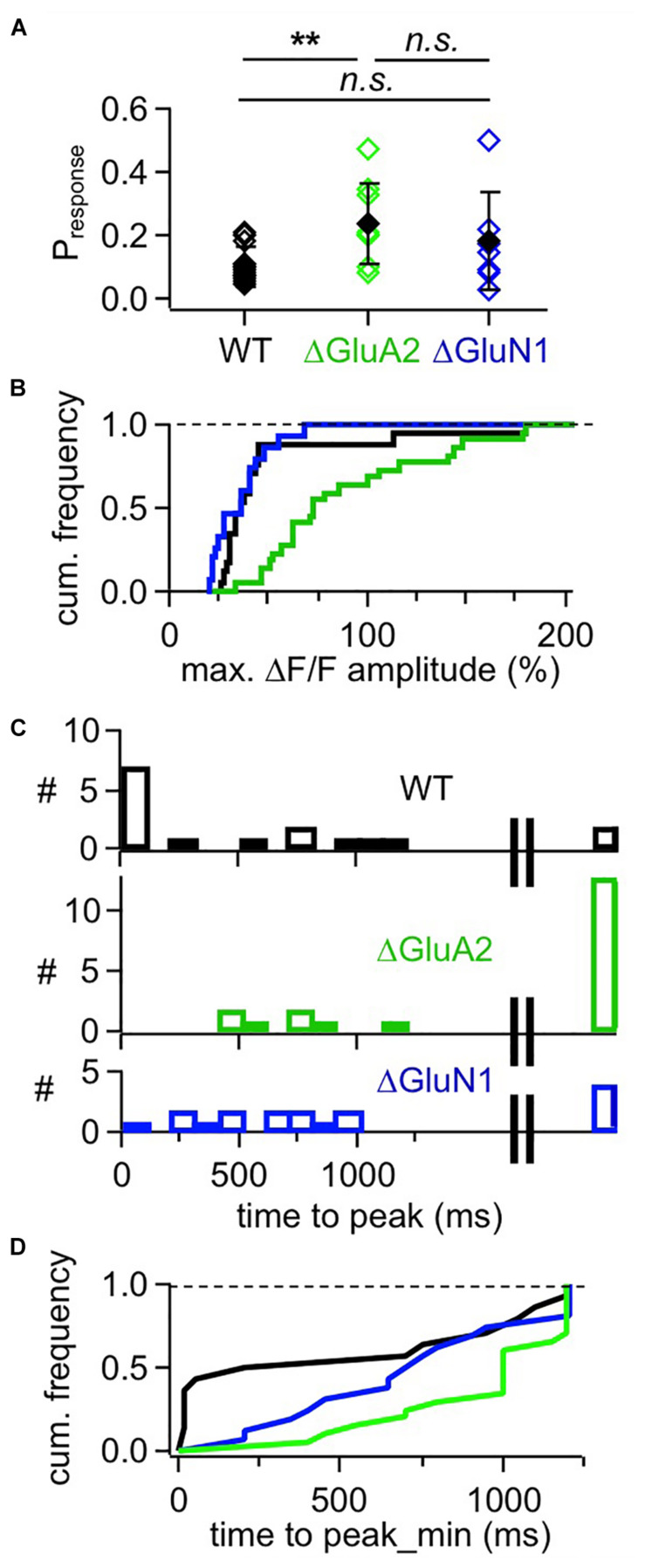
Cumulative analyses of responses in adult mouse WT, ΔGluA2 and ΔGluN1 GCs. **(A)** Estimated likelihood of observing a (ΔF/F)_syn_ response upon glomerular stimulation (“response probability,” calculated as number of responses divided by number of stimulations). Pairwise comparison (Mann-Whitney test) and Bonferroni: *P*_*r*_ WT vs. ΔGluA2: *P* = 0.0018, ΔGluA2 vs. ΔGluN1: *P* = 0.42, WT vs. ΔGluN1: *P* = 0.51. **(B)** Frequency distribution of (ΔF/F)_syn_ response amplitudes for the three GC groups. WT is different from ΔGluA2, but not from ΔGluN1 (*P* < 0.001 and *P* = 0.16, Kolmogorov-Smirnov). **(C)** Distributions of (ΔF/F)_syn_ response time to peak for the three GC groups. The rightmost bar shows the responses where the peak might not yet have been reached within the scanning time window (up to 1,200 ms post stimulation). **(D)** Frequency distribution of (ΔF/F)_syn_ response time to peak for the three GC groups, with peaks possibly beyond scan window set to 1,200 ms (minimal time to peak). WT is different from ΔGluA2, but not from ΔGluN1 (*P* = 0.02 and *P* = 0.09, Kolmogorov-Smirnov test).

Postsynaptic Ca^2 +^ transients in WT adult mouse GC spines were also strictly localized to spine heads and occurred with a rather low probability upon glomerular stimulation, even though compound EPSPs were readily recorded at the soma (see Figure 3B), rendering the set of recorded individual responses rather small (estimated response probability *P*_*r*_ 0.10 ± 0.06, *n* = 13 spines; Figures 3A, 4A–C, see section “Methods”). While some responses rose rather quickly (e.g., top left response in Figure 3B, distribution in Figures 4B,C), in most cases the peak amplitude of these (ΔF/F)_syn_ signals occurred several 100 ms later (average time to peak (TTP) 420 ± 440 ms, beginning with 20 ms, *n* = 14 events; 2 events with peak beyond scan time of 1,000 ms; if these are included with the end of scan as peak time: TTP_min_ = 540 ± 510 ms). TTP was uncorrelated to peak amplitude (*r* = 0.32, *P* = 0.11, *n* = 14). Such slowly rising signals were never observed in the adjacent dendrite. We also recorded responses to single backpropagating APs (ΔF/F)_sAP_ in 9 of the 13 spines, with mean rise times of 15 ± 4 ms (see also Stroh et al., 2012).

This slow rise was unrelated to Ca^2 +^ entry via NMDARs, since similarly slow signals occurred in ΔGluN1 GC spines with reduced Ca^2 +^ entry (Figures 3B, 4A–C; ΔGluN1 TTP 580 ± 260 ms, beginning with 200 ms, *n* = 13 events; 4 events with peak beyond scan, TTP_min_ = 740 ± 350 ms; no significant difference to WT TTP_min_: Kolmogorov-Smirnov test *P* = 0.09). The probability to observe such events in ΔGluN1 GCs (*P*_*r*_ = 0.18 ± 0.15, *n* = 7 spines) was also not significantly different from WT.

Additional Ca^2 +^ entry due to GluA2 deletion, however, resulted in larger ΔF/Fs that rose even more slowly than in WT spines (Figures 3B, 4A–C; ΔGluA2 TTP 680 ± 250 ms, beginning with 400 ms, *n* = 7 events; 13 events with peak beyond scan; all events: TTP_min_ = 960 ± 270 ms: Kolmogorov-Smirnov test *P* < 0.02 vs. WT). The probability of such events was significantly higher than in WT (*P*_*r*_ = 0.24 ± 0.13, *n* = 8 spines, *P* = 0.006), possibly due to their improved detectability because of larger ΔF/F amplitudes. Such a slow evolution in postsynaptic Ca^2 +^ should also be reflected in the strength and time course of reciprocal GABA release. Indeed, it was shown previously in recordings of mitral cell hyperpolarizing potentials following a train of 20 APs at 50 Hz (a stimulation that efficiently activates reciprocal release from GCs; Figure 4 in Abraham et al., 2010), that ΔGluA2 resulted in a significantly stronger and almost two times longer mitral cell recurrent inhibition compared to WT. The relevant time scales match our time to peak data including the late peaks of (ΔF/F)_syn_ reported above (ΔGluA2: half duration t_1__/__2_ ∼1,150 ms; WT: t_1__/__2_ ∼600 ms). Conversely, ΔGluN1 did not exert a significant influence on the duration of recurrent inhibition and only a mild reduction on its amplitude, in line with the lack of a significant effect on TTP of (ΔF/F)_syn_ described above (Supplementary Information in Abraham et al., 2010).

## Discussion

### Relation Between Ca^2 +^ and Na^+^ Transients in the GC Spine Head and Asynchronous Release

Asynchronous release—i.e., release that happens later than the fast coupling of HVA presynaptic Ca^2 +^ currents to the release machinery (e.g., Kaeser and Regehr, 2014)—is a phenomenon known from many central synapses. It is often observed at repetitively stimulated synapses (Wen et al., 2013), which would also hold true for the classical dendrodendritic inhibition protocol, where voltage-clamped mitral cells are being depolarized for 20–50 ms and thus ongoing release of glutamate from mitral cells is likely to happen over dozens of ms and subsequent asynchronous release of GABA has been documented by many groups (see section “Introduction”). Thus it is at first surprising that local, unitary-like stimulation of GC spines by TPU would suffice to elicit asynchronous release, which we have recently documented (Lage-Rupprecht et al., 2020). However, the temporal extent of this asynchronous release was shorter than in the classical dendrodendritic inhibition experiments (maximal extent of ∼500 ms vs. >1 s) and therefore there might be additional mechanisms involved whenever GCs are activated more strongly.

The NMDAR-mediated Ca^2 +^ current into juvenile rat GC spines is expected to recede within less than 200 ms (e.g., Hestrin et al., 1990; Egger et al., 2005). It is thus on its own unlikely to mediate substantial asynchronous release far beyond the first 100 ms, even though the slow extrusion of Ca^2 +^ from the cytoplasm may contribute to delayed release (Egger and Stroh, 2009). To further unravel signaling downstream of the NMDAR and Na_v_ activation during the local spine spike, we investigated the time course of postsynaptic Na^+^ and Ca^2 +^ elevations with minimal exogenous buffering. Both ion species showed prolonged elevations for durations well compatible with asynchronous output.

Previously, somatic AP-mediated and postsynaptic Ca^2 +^ transients recorded with 100 μM OGB-1 were observed to decay with roughly equal half durations of 600 ms (except for the subpopulation of “slow spines” featuring transients with t_1__/__2_ > 1.5 s, which made up one third; Egger et al., 2005; Bywalez et al., 2015). Whereas here TPU-evoked transients were always substantially longer than AP-evoked transients in the same spine, on the order of 500 vs. 100 ms, dissociating physiological postsynaptic Ca^2 +^ dynamics from exogenous buffer effects. Interestingly, “slow spines” were not observed here, also not in an additional set of *n* = 12 spine responses that was excluded from the analysis because of inadvertently longer uncaging intervals. This observation might be explained by the existence of a Ca^2 +^-dependent extrusion mechanism, that is activated only by high levels of [Ca^2 +^]_i_ which are buffered away in the presence of 100 μM OGB-1 (such as Ca^2 +^-ATPases, Na^+^/Ca^2 +^ exchangers or mitochondrial Ca^2 +^ uniporters, e.g., Sabatini et al., 2002; Chamberland et al., 2019).

In particular, there was a substantial and long-lasting postsynaptic elevation of Na^+^. This detected Δ[Na^+^]_i_ is ∼20-fold higher than what could be extrapolated for a single backpropagating GC AP from responses to train stimulation (∼10 vs. ∼0.4 mM, see Figure 2A). Thus there must be substantial postsynaptic Na^+^ entry on top of local Na_v_ activation, in line with earlier reports of Na^+^ signals in response to suprathreshold synaptic stimulation in hippocampal pyramidal neurons (Rose and Konnerth, 2001). In GC spines, the average time course of Δ[Na^+^]_i_ showed a plateau-like phase of >500 ms, very much unlike recent observations of single synaptic Na^+^ transients in spines of hippocampal pyramidal neurons which decayed within 20 ms—but were of a similar magnitude (∼5 mM; Miyazaki and Ross, 2017). While the slow decay of the GC spine Δ[Na^+^]_i_ might be explained to some extent by the diffusive barrier provided by the high neck resistance (predicted as ≥1 GΩ, Bywalez et al., 2015), the origin of the [Na^+^]_i_ plateau requires a persistent Na^+^ entry that outlasts AMPAR/NMDAR activation. It thus might indeed be related to extended local TRPC1/4 activation downstream of NMDAR activation, since there was no global plateau current in ΔGluN1 GCs (Stroh et al., 2012).

Alternatively or in addition, the [Na^+^]_i_ plateau might correspond to a local UP state, i.e., a local plateau potential [which are known to occur in dendrites of e.g., prefrontal pyramidal cells or striatal spiny neurons (Milojkovic et al., 2005; Plotkin et al., 2011)] in the GC spine which could cause TRPC1/4 activation and thus ongoing local influx of Ca^2 +^ sufficient to trigger recurrent release. This influx should also happen close to the release machinery, since buffering of GC Ca^2 +^ by EGTA had no effect on asynchronous release (Isaacson, 2001). Such local UP states or plateau potentials might not be evident in GC somatic membrane potential recordings due to substantial filtering by the large spine neck resistance, even though preliminary simulations of UP states in GC spines show that such plateaus would undergo considerably less filtering that spine spikes because of their slow kinetics (Aghvami and Egger, unpublished observations). So far, evidence for plateau-like states within GC spines could not be gathered from our somatic current clamp recordings; spine recordings with advanced voltage-sensitive dyes would be required to further elucidate this issue. Increased [Na^+^]_i_ within the observed regime might also provide positive feedback to NMDARs via an upregulation of NMDAR Ca^2 +^ currents by the Src kinase (Yu et al., 1997; Yu and Salter, 1998) and thus cause persistent Ca^2 +^ and Na^+^ entry. Further experiments are required to unravel such interactions.

The large size of Δ[Na^+^]_i_ amplitudes in the adjacent dendrite is unexpected, since Na^+^ diffusion from the spine into the dendrite should result in a substantial drop in concentration (e.g., Miyazaki and Ross, 2017) and GC spine necks are particularly long (∼6 μm in our sample, Figure 2C). Possible explanations for this observation include the large volume of GC spine heads (similar radius as the dendrite, Egger and Stroh, 2009), weak Na^+^ extrusion from the spine head and neck, or that the source of the synaptically triggered persistent Na^+^ entry mechanism postulated above is present and activated also within the dendritic shaft. In any case, dendritic Na_v_ channels (e.g., Egger et al., 2003; Nunes and Kuner, 2018) are unlikely to contribute to this signal since we have shown previously that the postsynaptic spine depolarization undergoes strong attenuation and thus both dendritic Na_v_ and Ca_v_ channels will not become activated (Bywalez et al., 2015)—unless several spines receive coincident inputs (Müller and Egger, 2020).

### Postsynaptic Ca^2 +^ Signals in Adult Mouse vs. Juvenile Rat

Endogenous Ca^2 +^ buffering and extrusion, postsynaptic ΔCa^2 +^ amplitudes and synaptic AP-evoked Ca^2 +^ signals and long-lasting depolarizations are similar in adult mouse GCs as in juvenile rat GCs (Egger et al., 2005; Egger, 2008; Egger and Stroh, 2009; Abraham et al., 2010; Stroh et al., 2012). However, a more detailed analysis of the synaptic responses showed also two striking differences. First, we observed a strongly reduced release probability *P*_*r*_ (0.1 vs. 0.5). This effect might be due to maturation of the bulbar network, since in rats the strength of dendrodendritic inhibition was reported to decline steeply between PND15 and PND20 (Dietz et al., 2011). While this *P*_*r*_ value is no more than an estimate due to the small number of recorded responses and substantial noise in some experiments, we observed similar values also for recordings from ΔGluN1 and ΔGluA2 GCs, with the slight increase for ΔGluA2 possibly explained by the improved detectability of signals in these cells.

Second, yet more strikingly, we observed a broad variability in signal onset and rise. In all three types of GCs, there was a subset of signals with an apparently delayed onset and a very slow evolution within hundreds of milliseconds up to seconds. These features were unchanged between WT and ΔGluN1, so NMDARs are not required to generate such responses. Rather, the ΔGluA2 GCs showed an increased fraction of such slow signals, indicating that Ca^2 +^-permeable AMPARs can also feed into this mechanism. This effect could be mediated perhaps by enhancing postsynaptic Ca^2 +^ induced Ca^2 +^ release (CICR) that we have previously shown to also occur in rat GC spines (Egger et al., 2005). Such a Ca^2 +^-dependent mechanism might also be supported by slow extrusion (Egger and Stroh, 2009). Together with the current observations these data are consistent with voltage-gated Ca^2 +^ channels or Ca^2 +^-permeable AMPARs triggering CICR, rather than NMDARs, as was also observed in other neuron types (e.g., Chávez et al., 2006; Plotkin et al., 2013). In any case, the respective mechanism is also likely to undergo developmental upregulation since a delayed and extended postsynaptic Ca^2 +^ rise was not observed in young rat GC spines. In adult mice, somatic GC Ca^2 +^ responses to odorants were also reported to show a high variability with regard to onset and rise (Wienisch and Murthy, 2016).

Intriguingly, apparently slowly rising signals in GC spines may also be of presynaptic origin, e.g., due to late firing of principal neurons in response to glomerular stimulation (Kapoor and Urban, 2006; Gire et al., 2012; Giridhar and Urban, 2012). In our experiments, this source might also contribute in the wake of glomerular stimulation; a correlation with late EPSPs is difficult to test because of the high spontaneous activity and low number of responses in our recordings. However, such delayed presynaptic activity is probably not a main source of slowly rising signals in our data set, since an enhancement of presynaptic signal contributions specifically in ΔGluA2 GCs appears rather unlikely.

As already implied by this possibility of delayed presynaptic inputs, slow signals in OB networks are not restricted to GC-mediated recurrent inhibition; representations of olfactory stimuli in general are known to evolve over long time scales of hundreds to thousands of ms (Friedrich and Laurent, 2001; Uchida and Mainen, 2003; Abraham et al., 2004, 2010; Rinberg et al., 2006; Gschwend et al., 2015). Aside from the original notion that these time scales are required for decorrelation of principal neuron activity, persistent representations might also be involved in the formation of odor after-images (Patterson et al., 2013).

With regard to further functions of slow signals, they are at first glance unlikely to play a direct role during odor discrimination or background segregation, since these discriminations usually occur within considerably less than 500 ms, even for difficult mixtures and/or many components (Abraham et al., 2004, 2010; Kepecs et al., 2005; Rokni et al., 2014; Bhattacharjee et al., 2019). Rather, slow signals may be involved in learning and plasticity, also during learning of the mixture discrimination task (Abraham et al., 2004, 2010; Gschwend et al., 2015).

Aberrant slow signals due to pathological changes (extended or reduced asynchronous release) would thus be expected to disrupt plasticity induction. Indeed, several pathologies such as Alzheimer’s disease have been associated with enhanced asynchronous release (reviewed in Kaeser and Regehr, 2014), for example at the neuromuscular junction, and interestingly also for fast-spiking interneurons in epileptic foci in both human and rat (Jiang et al., 2012). However, so far no loss of function has been observed for the ΔGluA2 modification. Pathological or other modulations of asynchronous release from granule cells might also influence both slow and fast network oscillations, i.e., the respiration coupled θ and probably more importantly γ rhythm that GCs have been associated with (e.g., Fukunaga et al., 2014). Such interactions between asynchronous release and rhythmic activity have been demonstrated in other GABAergic interneurons, including hippocampal cholecystokinin-positive basket interneurons that are crucially involved in generation of the hippocampal theta rhythm (e.g., Hefft and Jonas, 2005), and in cortical parvalbumin-positive GABAergic neurons that power γ oscillations (e.g., Traub et al., 1998). Interestingly, increased asynchronous release of GABA might reduce the ability of these PV+ neurons to sustain γ and has been proposed as one possible mechanism for uncoupling in schizophrenia (Volman et al., 2011).

In conclusion, we find that several mechanisms such as delayed and slowly evolving excitation, slow removal of Ca^2 +^ and perhaps extended local postsynaptic depolarization as indicated by the persistent elevation of Na^+^ may feed into asynchronous GC spine output. Since on the other hand there is also a fast, synchronous component of reciprocal release (Halabisky et al., 2000; Lage-Rupprecht et al., 2020), GC spines are obviously capable of parallel processing on multiple time scales, a property that appears to be further refined with maturation.

## Data Availability Statement

The raw data supporting the conclusions of this article will be made available by the authors, without undue reservation.

## Ethics Statement

Ethical review and approval was not required for the animal study because according to German animal welfare legislation, our experiments in acute brain slices of rats and mice are classified as *in vitro* and do not require the approval of an ethics committee. We are, however, monitored and certified by the authorities with regard to animal handling and the preparation process, which involves anesthesia and decapitation.

## Author Contributions

Rat granule cell Ca^2 +^ imaging was performed by VL-R, rat granule cell Na^+^ imaging by TOJ, mouse viral injections by NA and mouse granule cell Ca^2 +^ imaging by VE. VL-R, TOJ, and VE analyzed the data. VE, NA, and CR designed the research. VE wrote the manuscript. All authors except for TOJ contributed to editing the manuscript.

## Conflict of Interest

The authors declare that the research was conducted in the absence of any commercial or financial relationships that could be construed as a potential conflict of interest.
